# Knowledge, Attitude, and Perception of Urban Indian population towards Corona Virus Vaccination

**DOI:** 10.4314/ejhs.v35i5.9

**Published:** 2025-09

**Authors:** Rama Parthasrathy, Jayakumar Rajagopal, M Gunasekar

**Affiliations:** 1 Department of pharmacy practice, PSG College of Pharmacy, Coimbatore, India 641004; 2 Department of Respiratory Medicine, PSG Hospitals, Coimbatore – 641004

**Keywords:** Knowledge, Attitudes, Perceptions, COVID-19 vaccine, Acceptance

## Abstract

**Background:**

In COVID-19 vaccination, the government faced considerable challenges in encouraging people to get vaccinated due to misinformation, vaccine hesitancy, and communication gaps. This study aimed to assess the knowledge, attitudes, and perceptions towards COVID-19 vaccination among the urban population of Coimbatore district, Tamil Nadu.

**Methods:**

A cross-sectional, observational, questionnaire-based study was conducted among the urban population of Coimbatore district, Tamil Nadu. The questionnaire, adapted from a previous study, was translated into both English and Tamil and comprised four sections: socio-demographics, knowledge, attitudes, and perceptions. Data were collected through both offline (printed) and online (Google Forms) methods over a one-year period prior to the widespread rollout of mass COVID-19 vaccination programs in India. A total of 402 participants were enrolled, and responses were analyzed using frequencies and percentages. A p-value < 0.05 was considered statistically significant.

**Results:**

The study revealed that 51.24% of participants demonstrated high knowledge, 69.65% had a positive attitude, and 85.82% showed a generally favorable perception toward vaccination. The mean knowledge and attitude scores were 2.45±1.16 and 9.35±3.28, respectively. About 54% of participants agreed that vaccines are safe, while 51% believed that vaccination increased allergic reactions. A majority (57.96%) relied on mass media, alongside social media, as their main source of vaccine-related information. Logistic regression analysis indicated that gender, education, and occupation were not significant predictors of knowledge, attitude, or perception. Age, however, emerged as a significant predictor.

**Conclusion:**

The study confirmed that participants had reasonable knowledge and favorable attitudes and perceptions regarding COVID-19 vaccination. Nevertheless, misinformation and unverified content on social media contributed to hesitancy among some individuals.

## Introduction

COVID-19 is widely recognized as a respiratory disease that presents as an acute upper and/or lower respiratory tract infection with varying levels of severity, progressing gradually from cough and fever to the life-threatening condition of pneumonia (1). Initially, the primary goal of disease control was to prevent transmission between individuals. Vaccination was later introduced as a strategy to achieve herd immunity against the disease (2). However, the government encountered significant challenges in promoting COVID-19 vaccination due to misinformation, vaccine hesitancy, and communication gaps (3). The acceptance and coverage of vaccines vary across time, place, and population behavior (4).

Several studies have shown that vaccine hesitancy is influenced by concerns over long-term side effects, the rapid development and approval of vaccines, and general distrust in pharmaceutical companies and government health agencies. Demographic factors such as age, gender, and education also play a role in shaping acceptance of the COVID-19 vaccine (5). Moreover, after prolonged lockdowns and restrictions, many individuals experienced pandemic fatigue and showed reduced interest in vaccination efforts (6).

Effective communication on vaccine benefits and safety has been critical; however, language barriers, varying literacy levels, and differing levels of trust in authorities have complicated these efforts (7). Identifying the root causes of vaccine hesitancy in specific populations is essential to designing targeted interventions that enhance acceptance. From an urban perspective, such research can help strengthen public trust in health authorities and vaccination programs. Against this background, this study aimed to assess the knowledge, attitudes, and perceptions toward COVID-19 vaccination among the urban population of Coimbatore district, Tamil Nadu.

## Methods

A cross-sectional observational study was conducted among the general population aged 18–75 years in Peelamedu and surrounding areas of Coimbatore city. The study adhered to ethical principles and institutional guidelines, and approval was obtained from the Institutional Ethics Committee (proposal number: 2021/074). Informed consent was secured from all participants. Data were collected over a one-year period, before the widespread rollout of mass COVID-19 vaccination programs in India, at a time when public awareness, vaccine availability, and official communication strategies were still at an early stage.

The questionnaire, adapted from Islam MS et al. (2021) (8), was translated into English and Tamil and consisted of four sections: socio-demographics, knowledge, attitudes, and perceptions. Data were collected through both offline (printed forms) and online (Google Forms) methods. The sample size, calculated using the formula n = z^2^PQ/e^2^ (with Z=1.96, P=50%, Q=50%, at a 95% confidence interval and 5% margin of error), was 383. Participants were randomly recruited from approximately 97,233 residents of Peelamedu. Extensive responses via Google Forms increased the sample size to 402.

The inclusion criteria were individuals aged 19–75 years who provided consent. Exclusion criteria were participants under 18 years of age or those unwilling to consent. Socio-demographic information (age, gender, education, occupation) was collected, alongside participants' vaccination history and prior COVID-19 infection.

To assess knowledge, attitudes, and perceptions, 18 structured questions were used: 7 for knowledge, 6 for attitudes, and 6 for perceptions.

**Knowledge**: 4 dichotomous (Yes/No) questions; “Yes” = 1, “No” = 0. Scores ranged from 0–4, with higher scores indicating greater knowledge.

**Attitudes**: 6 items measured on a 3-point Likert scale (0=Disagree, 1=Undecided, 2=Agree). Scores ranged from 0–12, with higher scores reflecting more positive attitudes.

**Perceptions**: 6 items, including 4 Yes/No questions and 2 related to vaccine application.

Data analysis was conducted using Microsoft Excel and SPSS (version 20). Excel was used for data entry, editing, and coding, after which the data were imported into SPSS. Descriptive statistics (frequencies, percentages, means, and standard deviations) and regression analyses (logistic and multiple linear regression) were applied. A p-value < 0.05 was considered statistically significant.

## Results

A total of 402 participants were surveyed, with a majority being male (63.43%) and aged 18-29 years (62.44%); most were students (62.44%). Regarding education, 61.94% had completed undergraduate studies and 21.14% were postgraduates. Vaccination history revealed that 73.1% had received all necessary vaccines, while 88.1% reported prior COVID-19 infection. Table 1 presents the demographic characteristics of study participants

**Table 1:** Socio-demographics and KAP score of study participants

Variables	N=402(%)	Knowledge Score Mean (SD)	Attitude Score Mean (SD)	Perception Score Mean (SD)
**Age**				
18-29	251(62.4)	2.29(1.19)	9.29 (3.16)	4.00(1.23)
30-39	62(15.4)	2.60 (1.03)	10.02 (2.89)	4.47(1.33)
40-49	47(11.7)	2.74(1.11)	9.30 (3.69)	4.17(1.29)
50-60	38(9.5)	2.76(1.10)	8.61 (4.10)	3.66(1.48)
>60	4(1.0)	3.25 (0.50)	10.25 (1.25)	4.50(1.29)
**Gender**				
Male	255(63.4)	2.55 (1.14)	9.64 (3.20)	4.13(1.66)
Female	147(36.6)	2.28 (1.18)	8.84 (3.37)	3.96(1.66)
**Education**				
None	26(6.5)	2 (1.02)	6.46 (3.94)	3.23(1.19)
School	42(10.5)	2.31 (1.18)	9.26 (3.79)	3.86(1.19)
Undergraduate	249(61.9)	2.47 (1.17)	9.74 (2.83)	4.16(1.19)
Postgraduate	85(21.1)	2.58 (1.14)	9.11 (3.61)	4.15(1.19)
**Occupation**				
Unemployed	39(9.7)	2.13 (1.08)	9.26 (3.49)	3.79(1.39)
Student	162(40.3)	2.24 (1.18)	9.20 (3.37)	3.94(1.39)
Housewife	30(7.5)	2.27 (1.05)	7.70 (3.75)	3.87(1.39)
General worker	125(31.0)	2.68 (1.13)	9.74 (3.05)	4.28(1.39)
Healthcare worker	46(11.4)	2.93 (1.06)	9.91 (2.76)	4.30(1.39)
**All necessary vaccines**				
Yes	294(73.5)	2.59 (1.15)	9.69 (3.03)	4.19(1.70)
No	106(26.5)	2.06 (1.07)	8.45 (3.75)	3.74(1.70)
**Previously infected with corona virus**				
Yes	48(11.9)	2.77(1.17)	10.44(2.77)	4.46(1.44)
No	354(88.1)	2.41(1.14)	9.2(3.33)	4.01(1.44)

Knowledge scores were higher among participants with higher education (2.58±1.14) and healthcare workers (2.93±1.06). Males had a higher mean knowledge score (2.55±1.14), and participants over 60 years had the highest mean score (3.25±0.50). Participants with no formal education had the lowest mean score (2.00±1.02). Attitude scores were also higher among males (9.64±3.20), those with higher education (9.74±2.83), and participants with prior COVID-19 infection (10.44±2.77). Housewives had the lowest mean attitude score (7.70±3.75). The average perception score was 4.07±0.27 out of 6, with higher scores among males, the elderly, educated participants, and healthcare workers.

Knowledge assessment showed that 97% were aware of COVID-19 vaccines. More than half (53%) knew about vaccine effectiveness, 51% believed vaccines caused allergic reactions, and 56.7% were unaware of possible side effects. Most participants (57.96%) cited mass media, along with social media, as their primary information sources. Overall, 51.24% demonstrated high knowledge, though the average knowledge level across participants was moderate (49%) as detailed in Table 2.

**Table 2:** Assessment of Knowledge using questionnaire among study participants

Questions (n=402)	Yes n (%)	No n (%)
**Do you know about COVID-19 vaccine?**	390 (97.0)	12 (3.0)
**Do you know about the effectiveness of COVID-19 vaccine?**	213 (53.0)	189 (47.0)
**Does vaccination increase allergic reactions?**	207 (51.5)	195 (48.5)
**Do you know any side effects caused by COVID-19 vaccine?**	174 (43.3)	228 (56.7)
**Social media is a source of knowledge about the COVID-19 vaccine**	232(57.96)	170(42.44)

**Figure 1:**
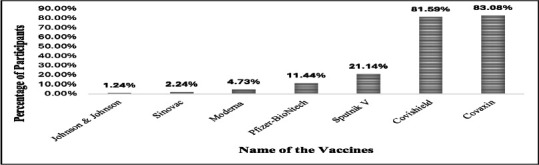
Percentage of COVID-19 vaccines known by the participants

**Figure 2:**
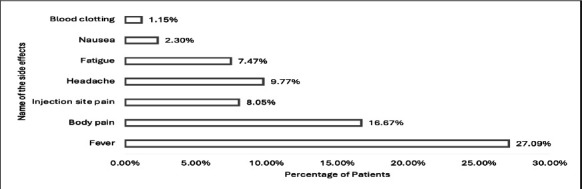
Participants Reported Side effects caused by COVID-19 vaccine

Awareness of Covaxin (83%) and Covishield (81%) was high, but knowledge of other vaccines was limited: Sputnik (21.14%), Pfizer (11.44%), Moderna (4.73%), Sinovac (2.24%), and Johnson & Johnson (1.24%)as shown in Figure 1. Reported side effects included fever and body pain (27.09%), explained in the Figure 2.

Attitude assessment revealed that 54% agreed COVID-19 vaccines were safe, 74% agreed they should be vaccinated, and 70% reported no hesitation in receiving the vaccine. About 73% would recommend vaccination to others, and 67% agreed that immunization was necessary to reduce COVID-19 incidence. Nearly 69.65% showed favorable attitudes overall.

Perceptions varied: 71.4% trusted natural or traditional remedies over vaccines, and 71.1% believed COVID-19 could be controlled with preventive measures alone. Most participants (61%) believed healthcare workers should be prioritized, while 66% supported mandatory vaccination. A majority (77%) considered vaccines effective, and 59% would pay for vaccination if not provided free. Table 3 and Table 4 present the assessment of attitude and perception among the study participants.

**Table 3:** Assessment of Attitude using questionnaire among study participants

Questions (n=402)	Disagree n (%)	Neutral n (%)	Agree n (%)
**The newly discovered Covid-19 vaccines are safe?**	27 (6.7)	157 (39.1)	218 (54.2)
**The Covid-19 vaccines are essential for us?**	30 (7.5)	75 (18.7)	297 (73.9)
**I will take the COVID-19 vaccine without any hesitation?**	65 (16.2)	54 (13.4)	283 (70.4)
**I will also encourage my family/friends'/relatives to get vaccinated?**	84 (20.9)	22 (5.5)	296 (73.6)
**It is not possible to reduce the incidence of COVID-19 without vaccination?**	47(11.7)	86 (21.4)	269 (66.9)
**I think that the approval of the vaccine from government guarantees its safety?**	34 (8.5)	99 (24.6)	269 (66.9)

“a” indicates the score Disagree =0; b indicates the score Neutral=1; c indicates the score agree =2. d indicates the average knowledge score among participants.

**Table 4:** Assessment of Perception using questionnaire among study participants

Questions (n=402)	Yes (a) n (%)	No (b) n (%)
**I believe in natural or traditional remedies more than synthetic vaccines**	287 (71.4)	115 (28.6)
**Do you think that if everyone in the society maintains the preventive measures, the COVID-19 pandemic can be eradicated without vaccination?**	286 (71.1)	116 (28.9)
**Healthcare worker is supposed to be vaccinated first**	246 (61.2)	156(38.8)
**Everyone should have been vaccinated**	268 (66.7)	134(33.33)
**I trust the COVID vaccine is fighting effectively against the corona virus?**	311 (77.4)	91 (22.6)
**Would you buy the vaccine at your own expense if it was not provided free by the government?**	237 (59.0)	165 (41.0)

“a” indicates the score Yes =1; b indicates the score No=0. c indicates the average knowledge score among participants.

Regression analysis revealed age (t = -2.38, p = .018) and gender (t = 2.694, p = .007) as significant predictors of knowledge. However, multiple linear regression showed only weak associations between socio-demographic factors and attitudes (F(2,399) = 6.49, p = .002, R^2^ = 0.03) and no significant association with perceptions (F(1,400) = 1.55, p = .213, R^2^ = 0).

## Discussion

The severe impact of the COVID-19 pandemic on global health systems lasted for over two years before vaccines were developed and tested. Vaccination emerged as a viable solution, but its effectiveness relies heavily on public acceptance (9). This study provides insights into the knowledge, attitudes, and perceptions of the urban population in southern India.

Our findings align with studies by Islam MS et al. (2021) and Al-Zalfawi SM et al. (2021), where most participants were male, though this contrasts with Abdelhafiz AS et al. (2020), who reported a female majority (10, 11). The predominance of younger participants, largely students, parallels findings from Ferdous MZ et al. (2020), where students comprised 71.2% of respondents (12). Illiteracy, especially among the elderly, may hinder vaccine acceptance (13).

Knowledge levels in this study were moderate (49%), echoing Banik (2021), who emphasized the role of social media in shaping awareness (16). Improved communication and education, particularly in underserved areas, are crucial (17). Higher education and prior vaccination experience correlated with greater knowledge, consistent with previous studies (18).

Attitudes toward vaccination were largely positive and influenced by gender, consistent with findings from Wang (2020) and Samanta (2022) (19, 20). However, barriers such as misinformation, distrust, and safety concerns remain (21, 22). While nearly 70% expressed willingness to vaccinate, targeted campaigns are needed to reach the hesitant minority (23, 24). Contradictory results from Sudan (Babker AMA, 2024) highlight the complexity of these factors across regions.

Globally, vaccine hesitancy varies: 42.3% in Iran, 62.6% in India, and 66.4% in Egypt (25, 26). Natural remedies remain widely trusted, yet most participants supported universal vaccination and prioritized healthcare workers (27). Vaccine mistrust fueled by misinformation persists, with fewer participants willing to pay for vaccines compared to populations in Malaysia and Ecuador (28).

Regression analysis confirmed age and gender as predictors of knowledge, suggesting disparities in access to accurate information (29, 30). Early in the vaccination campaign, awareness was limited mainly to Covaxin and Covishield, reflecting the government's initial rollout strategy (31).

Larson HJ et al. identified determinants of hesitancy, including socioeconomic factors, communication environments, and health beliefs, while trust in healthcare and family influence improved acceptance (32). Similar findings from Sudan (Babker AMA, 2024) further underscore the universal nature of hesitancy despite contextual differences (33).

This study, conducted prior to mass vaccination, highlights vulnerable groups—such as housewives, the elderly, and the less educated—as requiring tailored communication. Culturally sensitive, community-based strategies are needed to dispel myths and build confidence.

Limitations include its cross-sectional design, reliance on self-reported data, and urban focus, limiting generalizability to rural populations. The timing of data collection, prior to mass vaccination, also constrains its applicability to evolving contexts with new variants and booster campaigns.

Nevertheless, this study contributes to understanding vaccine hesitancy and offers actionable insights for policymakers. Future longitudinal studies should assess changes in knowledge, attitudes, and perceptions over time, particularly after booster programs and variant outbreaks. Scalable approaches are also needed to evaluate the impact of misinformation across media platforms.

The study concluded that participants had a satisfactory degree of knowledge on COVID-19 vaccination, along with predominantly positive attitudes and perspectives. Nevertheless, despite this promising baseline, a segment of persons displayed vaccine hesitation. This hesitancy was significantly affected by the rise of misnformation and unverified content flowing on social media platforms.

Policymakers must implement region-specific campaigns, address myths, and collaborate with media to disseminate accurate information. Healthcare providers, as trusted educators, should communicate empathetically, use visual aids, and provide tailored support to reduce vaccine anxiety and improve acceptance.
